# Analysis of nanobody paratopes reveals greater diversity than classical antibodies

**DOI:** 10.1093/protein/gzy017

**Published:** 2018-07-25

**Authors:** Laura S Mitchell, Lucy J Colwell

**Affiliations:** Department of Chemistry, University of Cambridge, Lensfield Road, Cambridge, UK

**Keywords:** antibody, nanobody, paratope, single-domain antibody, VHH

## Abstract

Nanobodies (Nbs) are a class of antigen-binding protein derived from camelid immune systems, which achieve equivalent binding affinities and specificities to classical antibodies (Abs) despite being comprised of only a single variable domain. Here, we use a data set of 156 unique Nb:antigen complex structures to characterize Nb–antigen binding and draw comparison to a set of 156 unique Ab:antigen structures. We analyse residue composition and interactions at the antigen interface, together with structural features of the paratopes of both data sets. Our analysis finds that the set of Nb structures displays much greater paratope diversity, in terms of the structural segments involved in the paratope, the residues used at these positions to contact the antigen and furthermore the type of contacts made with the antigen. Our findings suggest a different relationship between contact propensity and sequence variability from that observed for Ab VH domains. The distinction between sequence positions that control interaction specificity and those that form the domain scaffold is much less clear-cut for Nbs, and furthermore H3 loop positions play a much more dominant role in determining interaction specificity.

## Introduction

**Fig. 1:**
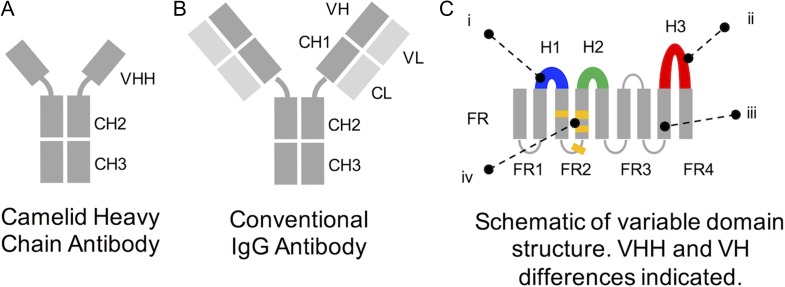
Schematic overview of VHH and VH domains. VHHs (Nbs) are the variable domain derived from camelid HcAbs (**A**), whereas VHs are the heavy chain variable domain derived from classical Abs (**B**). These homologous immunoglobulin domains have four key differences, represented in (**C**): (i) Nb H1 loops tend not to fit canonical conformations seen in Ab H1 loops, (ii) Nb H3 loops can be longer in length, (iii) Nb framework regions (FRs) are more conserved in both sequence and structure than Ab FRs and (iv) Nb FR2 have four solubility-enhancing mutations that facilitate stability without a partnering VL domain.

Nanobodies (Nbs) are small single-domain proteins found in the immune systems of camelid species (Hamers-Casterman *et al.*, 1993). Like antibodies (Abs), they specifically bind particular targets within complex environments, such as tumour surfaces, cell lysates and intracellular environments (Ries *et al.*, 2012; Chakravarty *et al.*, 2014; Kijanka *et al.*, 2015; Pleiner *et al.*, 2015). The Nb consists of the VHH domain from the homodimeric camelid heavy chain antibody (HcAb), which is homologous to the Ab heavy chain variable domain (VH domain). Isolated VHH domains are highly soluble and are fast emerging as an alternative Ab fragment for therapeutic, diagnostic and molecular research applications (De Meyer *et al.*, 2014). Nbs are thought to be functionally equivalent to full-length classical Abs; achieving nanomolar binding affinities and high specificities to seemingly any antigen the camelid immune system is challenged with (Muyldermans, 2013). These binding capabilities are achieved using a single variable domain with just three sequence-variable loops, compared to two variable domains of the VHVL binding subunit from monoclonal Abs (see Fig. 1). Nbs are distinguished by four critical framework mutations that enhance solubility, and H3 loops that are around 15% longer than Ab H3 loops (Sircar *et al.*, 2011; Muyldermans, 2013; Mitchell and Colwell, 2018).

Whilst these insights contribute to our understanding of the VHH domain itself, Nb:antigen interactions have not been well characterized. Do Nbs use similar strategies to Abs to create high affinity, highly specific interactions with their cognate antigen? The ability of any Ab to specifically interact with its cognate antigen arises through an evolutionary process, whereby antigen-binding molecules are selected from a large immune repertoire or library of Ab variants (Li *et al.*, 2004). Recently, the use of Nbs as crystallization chaperones has increased rapidly (Desmyter *et al.*, 2015), providing the structural data required for detailed analysis of the Nb:antigen interaction interface.

**Fig. 2:**
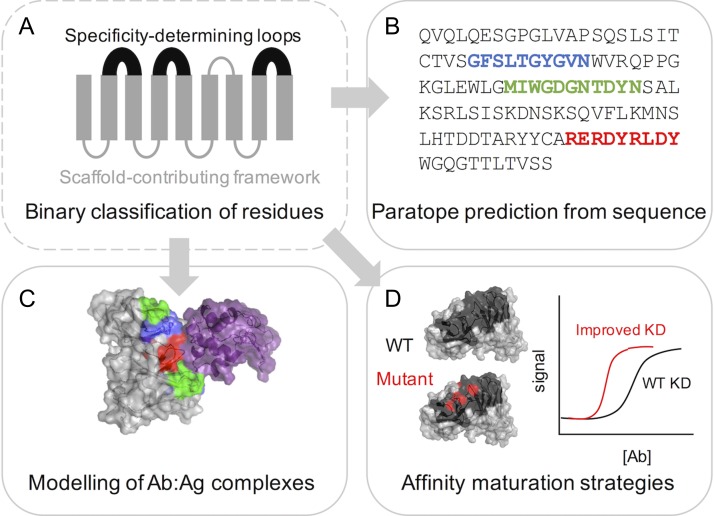
Variable domains in Abs can be conceptually divided into two regions. Structurally, the domain may be divided into framework and loop regions; or functionally it may be considered in terms of specificity-determining regions and scaffold regions (**A**). These distinctions are interchangeable in Abs, since the sequence-variable loop regions coincide with antigen contacts. This distinction is useful for (**B**) predicting paratopes from Ab sequences, (**C**) modelling Ab:antigen interactions and (**D**) engineering Ab properties.

For Abs, researchers have identified a natural division between the specificity-determining loop regions H1–3, which are highly variable and typically contact the antigen (Kunik *et al.*, 2012b), and the remaining framework regions (FRs) that form a scaffold providing support for the loops. This conceptual division, in which the structural and functional grouping of residues are equivalent, has inspired bioinformatic tools that identify the loop residues and predict antigen-binding residues from sequence, facilitating the modelling and engineering of Ab:antigen interactions (Fig. 2) (Chothia *et al.*, 1989; Kabat and Wu, 1991; Honegger and Plückthun, 2001; Abhinandan and Martin, 2008; Kunik *et al.*, 2012b; Dunbar and Deane, 2015).

In this manuscript, we use a set of 156 Nb:antigen co-crystal structures to characterize the Nb:antigen-binding interface. This data set includes Nbs derived by immunization strategies, selection from synthetic libraries and by protein engineering approaches. For each co-crystal structure, we define the paratope as those residues with atoms within 5 Å of the antigen structure. We compare these Nb paratopes to Ab VH domain paratopes extracted from 156 Ab:antigen co-crystal structures. We first classify individual paratopes by the segments they contain, to gauge the consensus among Nb and Ab paratopes. We then calculate class-averaged properties of each alignment position to understand the functional roles played by each residue. This allows us to distinguish scaffold residues from those directly involved in antigen interactions and highlights considerable differences between Nb and Ab paratopes. Finally, we analyse those amino acid pairs that make contact across the Nb:antigen and Ab:antigen interfaces, to establish chemical differences in the molecular recognition strategies exploited in each case.

## Materials and methods

### Data

We analyse 156 publicly available Nb:antigen and 156 Ab:antigen co-crystal structures (Berman *et al.*, 2000; Dunbar *et al.*, 2014), listed in Supplementary Tables S1 and S2. All Nbs and Abs have sequence-unique H3 loops, all antigens are proteins, and all structures have resolution at least 4.0 Å. The antigen diversity in the two sets is comparable; the 156 Nbs encode specificities to 126 distinct structural epitopes, while the 156 Abs encode specificities to 152 distinct structural epitopes. The VH and VHH domain sequences were extracted from pdb files and aligned using the AHo numbering scheme (Honegger and Plückthun, 2001) in which gaps are placed at loop centres, as implemented in the ANARCI webserver (Dunbar and Deane, 2015). The alignments used throughout the work are included as Figs. S1 and S2, together with an index for converting between alignment numbering, and AHo numbering, in Table S3. The ‘number of wild-type’ residues (nWT) at each alignment position is used as a measure of sequence conservation.

### Identification of paratope residues

To identify paratope and epitope residues from Nb and Ab co-crystal structures, we identify all residues for which the minimum atom distance to the nearest amino acid in the antigen chain(s) is <5 Å. Antigen-contacting residues in Nb and Ab chains were mapped to the abovementioned AHo sequence alignment for comparison.

### Contact profiles

For each Nb or Ab VH domain, we divide the structure into six segments (FR1, H1, FR2, H2, FR3 and H3) and score whether each segment contains any residue in contact with the antigen. The binary classifications for each segment were concatenated to generate a 6-position binary code for each structure, termed a ‘contact profile’. The FR4 region was omitted as very few contacts were found, reducing the number of possible contact profiles. We counted the number of unique contact profiles, and their corresponding frequencies in the Nb and Ab VH data sets.

### Calculation of solvent-accessible surface areas and paratope surface areas

Per-residue solvent-accessible surface areas (SASAs) were calculated for each VH and VHH domain using Lee and Richards’ algorithm (Lee and Richards, 1971), as implemented in the FreeSASA 2.0.1 Python module (Mitternacht, 2016), with default settings. These calculations are carried out for VHH domains in isolation, and for VH domains in complex with their cognate VL partners. The SASAs are then aligned to the AHo alignment, allowing class-averaged mean SASA per alignment position to be reported. The paratope surface areas are also calculated using FreeSASA, as the change in SASA upon complexation (paratope surface areas, ∆ASA) for each Nb/Ab. For Nbs, the total SASA of the Nb chain in complex with the antigen is subtracted from the total SASA of the isolated Nb. For Abs, VH and VL paratope surface areas are calculated individually, whilst keeping the two Ab chains intact for the antigen-‘isolated’ Ab.

### Extraction and alignment of pairwise residue contacts

Matrices (size 20 × 20) containing the frequency of residue–residue contacts across all 156 co-crystal structures are calculated for each aligned Nb/Ab position and plotted as heatmaps by summing matrices that correspond to specified segments of the alignment.

### Calculation of shape complementarity

Shape complementarity is calculated by the S_C_ method (Lawrence and Colman, 1993) using the S_C_ program (version 2.0) implemented in the Collaborative Computational Project, Number 4 (Winn *et al.*, 2011), using the default settings.

## Results and discussion

### Antigen-contacting regions

To characterize Nb–antigen binding, we first ask which regions of the Nb structure make contact with the antigen across Nb:antigen co-crystal structures. Previously, isolated examples have been used to suggest differences between the strategies used by Nbs and Abs. For example, De Genst *et al.* showed that unlike Abs, anti-lysozyme Nbs preferentially bind the active site cleft rather than the surface of the enzyme (De Genst *et al.*, 2006). Long Nb H3 loops can be inserted into crevices in active sites (e.g. anti-GPCR) (Rasmussen *et al.*, 2011). Cases where the Nb does not use one or two of the variable loops to contact the antigen have been noted (Decanniere *et al.*, 1999).

**Fig. 3:**
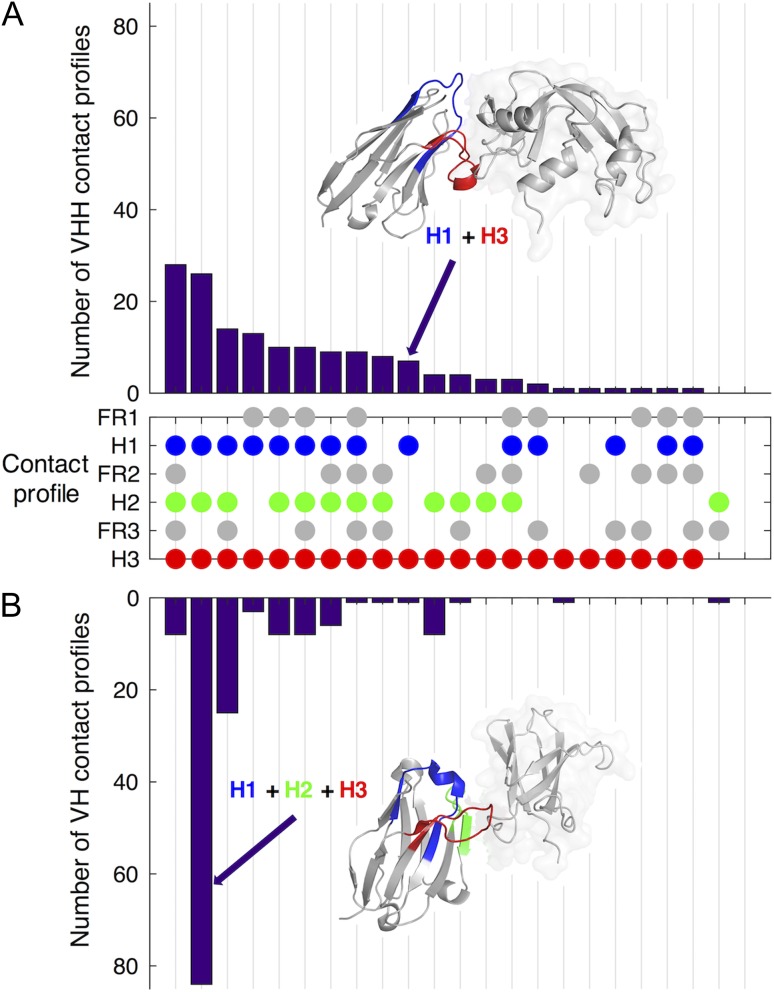
Contact profiles that describe the combination of interface residues used to contact the antigen by (**A**) Nbs and (**B**) Ab VH domains. The central dot matrix represents the range of observed contact profiles. Each profile is denoted by a vertical strip of dots, which indicates the combination of structural segments involved in a paratope. For example, the second profile with a blue, green and red dot indicates the class of Nbs and Abs that bind the antigen using only residues from loops H1–3. The frequency of each contact profile in the data set is indicated by the butterfly format bar charts. Note that while Abs often use just the three hypervariable loops H1–3 to contact the antigen (83/156), Nbs use a much more diverse distribution of possible contact profiles.

Are these differences exceptions, or examples of general phenomena? To address this question, we extract and analyse paratopes from sets of 156 Nb:antigen and Ab:antigen co-crystal structures. We classify each structure according to the combination of structural segments found in the paratope. We term each combination of antigen-contacting segments a ‘contact profile’ and report the distributions of contact profiles in Fig. 3. Strikingly, while a majority (53%) of Ab VH domains contact the antigen using loops H1–3, we find that only 16% of Nbs use loops H1–3, while overall Nbs use a far greater diversity of structural segment combinations to bind antigens. Among our data, there are 21 unique contact profiles observed in the Nb:antigen data set, compared to only 14 unique contact profiles in the VH:antigen data set. Almost one-third of Nb structures (30%) bind the antigen using only one or two of the three sequence-variable loops, compared to just 10% of Ab VH structures.

### Aligned contact propensity

**Fig. 4:**
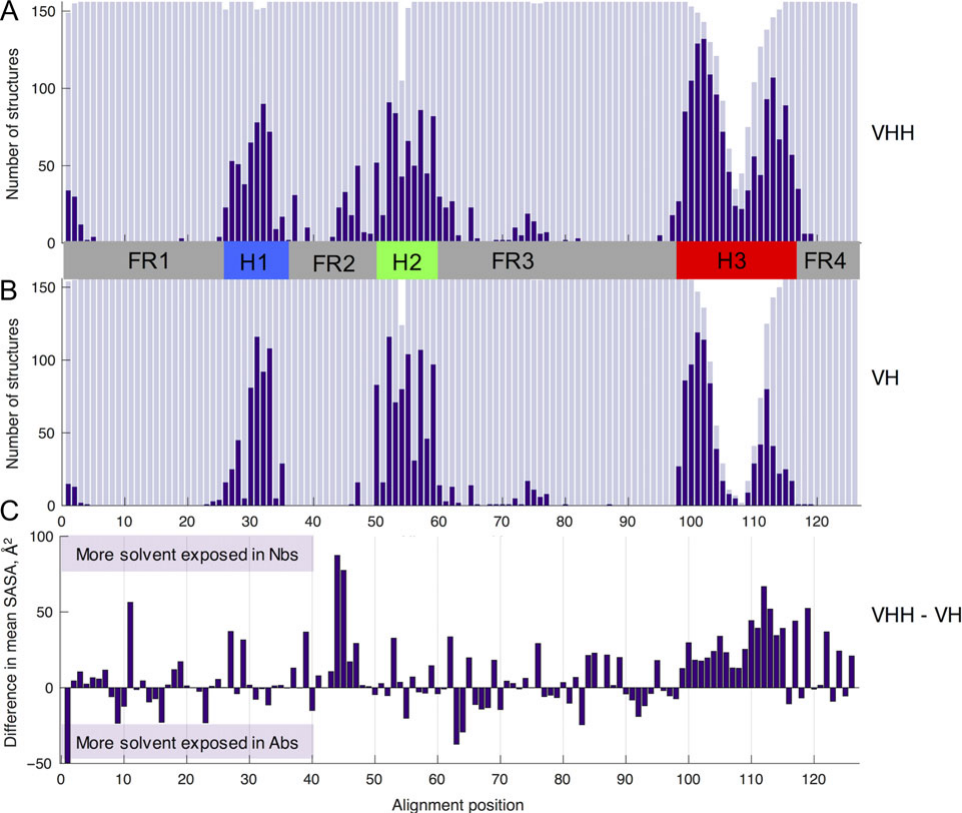
Antigen contact frequency for each of the 126 aligned positions in our data sets of Nb (**A**) and Ab (**B**) co-crystal structures, shown alongside the difference in mean SASA per aligned position (**C**). In (A) and (B), the number of sequences with a residue at each alignment position is plotted in light purple, and the number of structures that contact the antigen at each alignment position is overlaid in dark purple. Note the increased frequency of framework residues that contact the antigen in Nb VHH domains compared to Ab VH domains, which is linked to increased SASA in Nbs at these positions. The full distribution of SASAs for each aligned position, from which the difference in mean SASA was calculated is provided in Supplementary Fig. S3, data available at *PEDS* online.

To more closely examine how the antigen-contacting residues are distributed in Nbs, we use the frequency of antigen-contacting residues across all structures at each aligned sequence position to compute class-averaged ‘contact propensities’ for each position (Fig. 4). For both Nb and Ab co-crystal structures, the majority of paratope positions fall within aligned positions from the three loop regions. However, Nbs are able to draw their paratopes from a more diverse set of aligned positions, including positions at the N-terminal, in FR2 and adjacent to the loops. Greater contact propensity in Nbs across the FR2 correlates with solvent exposure of this region of beta sheet in the absence of the VL domain that obscures equivalent Ab positions from the antigen (Fig. 4C). Perhaps surprisingly, we also find lower antigen contact propensities across Nb H1 and H2 loops, compared to VH domains.

### Distinction between specificity-determining and scaffold positions

**Fig. 5:**
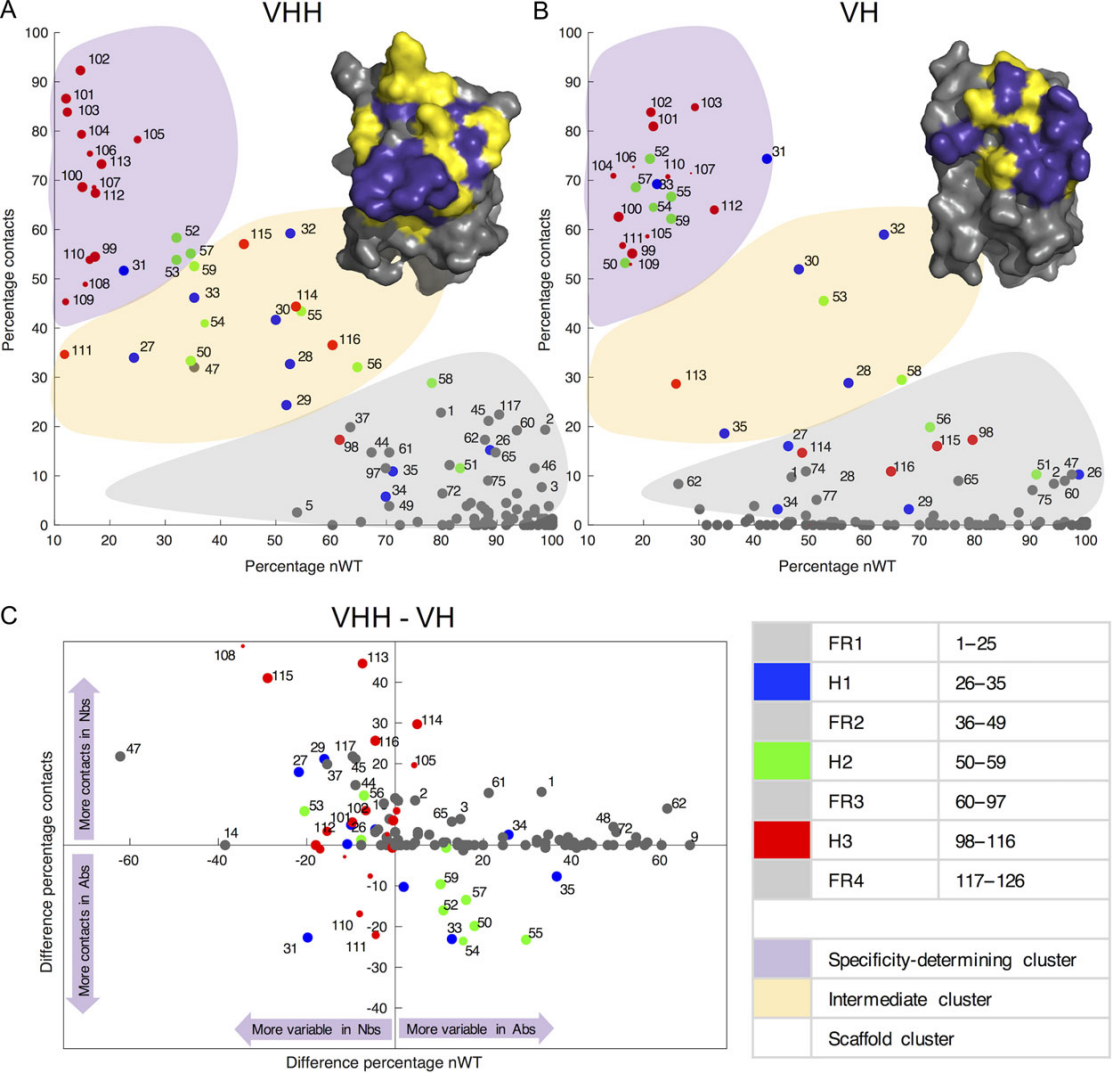
Scatter plots showing the relationship between sequence variability and contact propensity for each position of the AHo alignment, for (**A**) Nbs and (**B**) Ab VH domains. Points are labelled with alignment position, coloured according to structural segment of the molecule and sized according to the number of sequences with a residue at the alignment position. Inset representative Nb and Ab structures with surface rendering are oriented equivalently, and underlying residues are coloured according to the specificity-determining (purple), intermediate (yellow) or scaffold (grey) clusters they belong to. In the lower panel (**C**), the difference in conservation and contact propensity between equivalent VHH and VH alignment positions is plotted. A key to alignment positions and clusters is provided in the lower right panel.

Since the contact propensities differ between the two Ab classes for many aligned positions, we next ask whether their contribution to binding specificity also differs. We tease apart the functional roles of each aligned position by plotting contact propensity as a function of sequence conservation (Fig. 5A and B). The sequence conservation (nWT) is the proportion of aligned sequences that have the consensus amino acid residue at each sequence position. To aid interpretation we have shaded ‘guide clusters’ and mapped the positions onto representative VHH and VH structures. We posit that positions that display both high variability and high contact propensity encode antigen specificity (purple shading), in contrast to highly conserved positions that have low contact propensity (grey shading) (Padlan *et al.*, 1995). The anticipated negative correlation between conservation and contact propensity is observed in both data sets, though the correlation is much stronger in the Nb data set (*R *= −0.903) than the Ab data set (*R* = −0.651). This is in part caused by greater FR sequence variability in Ab VH domains (spread of grey FR positions along the *x*-axis in the Ab plot, Fig. 5B), despite the fact that these positions never contact the antigen. This greater FR sequence variability likely reflects the greater diversity in V genes from which Abs can be constructed (Muyldermans, 2013).

The most remarkable finding is that while the Ab specificity-determining cluster contains positions from all three loops (green, blue and red points), the specificity-determining cluster in Nbs is dominated by H3 loop (red) positions. Furthermore, Ab positions are largely divided between the purple ‘specificity-determining’ cluster and the grey ‘scaffold cluster’, while the yellow ‘intermediate cluster’ is sparsely populated (Fig. 5B). Such a clear distinction in the functional role of each position—between those that determine specificity, and those that act as a part of the scaffold—is much less apparent in the Nb data set. In Nbs, many positions fall in the intermediate yellow cluster, creating a more even spread along the line of best fit (Fig. 5A). The inset VHH and VH domain structures illustrate the mapping between these clusters and 3D structure. Figure 5C directly compares the properties of aligned positions between Abs and Nbs. We find that Nb H2 positions are generally less variable and have lower contact propensity, with the exception of positions 53 and 56. Here, Ab variability and contact propensity may be reduced by the frequent occurrence of Pro53 which packs between the loops and is not surface exposed, and also by Gly56 which has no side chain. The H3 positions are generally more variable in Nbs and more frequently contact the antigen, in particular positions 113–116 at the C-terminal end of the loop.

Intriguingly Fig. 5C shows that a number of FR positions lie above the line *y *= 0, and so have greater contact propensity in Nbs than Abs. Despite the fact that Nb FR sequences are generally more conserved (Mitchell and Colwell, 2018), a number of these positions display greater variability in Nbs, suggesting that they do contribute to antigen-binding specificity. These positions lie to the left of the line *x* = 0 and include positions 37, 44, 45, 47 and 117. Of these, positions 44–47 occur just before the H2 loop, on the VH–VL interface. In contrast, positions 1, 2, 61 and 62 have higher contact propensity in Nbs but are also more conserved, so likely contribute less to specificity.

### Paratope residue distributions

To understand how Nbs create specific binding, we examine how residue usage differs between Nb and Ab paratopes, and whether different residues, used at the same position, have different propensities to contact the antigen. In Fig. S4, in the Supplementary data available at *PEDS* online, the frequency of each residue at each alignment position is indicated by the size of the corresponding grey bubble. Overlaid red circles are sized according to the proportion of these residue types that are within 5 Å of the antigen in the co-crystal structure. Sequence-variable loops are marked in the sidebar; note the increased residue diversity (more small points) and greater contact propensity (red) in these regions. Across the Nb FRs, fewer, larger grey circles reflect increased sequence conservation. We observe that Nb FR position 37 tends to switch from the typical Phe to Tyr when it makes contact with the antigen (labelled ‘b’). Similarly, we note that position FR 47 (labelled ‘c’) is frequently Phe, Gly, Leu or Trp and is more likely to contact the antigen when it is Trp or Leu.

Across the H1 loop region, we find that Nbs use a greater range of residues to contact the antigen than Abs. Specifically, Nb H1 positions 26–35 sample 142/200 possible unique position–residue combinations, of which 110 contact the antigen. This compares to 90/200 for Abs, of which 67 contact the antigen. A key difference relates to the distribution of Tyr residues; in Nbs, Tyr contacts the antigen at nearly every H1 position (labelled Y1), compared to 5/10 Ab positions. We note that Phe29, conserved in Abs but not Nbs, is known to be a key residue in determining the presence of the Type 1 canonical H1 loop conformation. The hydrophobic side chain orients into the core of the domain, away from the antigen (Chothia *et al.*, 1989). In Nbs, the increased sequence diversity at position 29 likely contributes to the relative paucity of Type 1 H1 loops in our data set. We note a corresponding increase in paratope usage; position 29 (labelled ‘a’ in Supplementary Fig. S4, data available at *PEDS* online) makes contact with the antigen in 37/156 Nb structures, compared to just 5/156 Ab:antigen structures.

The Nb H2 loop has fewer total antigen-contacting residues over the 156 structures (579) than the Ab H2 loop (752). Despite this, the Nb H2 loop samples more residue–position combinations (146 bubbles, of which 122 contact the antigen) compared to the Ab H2 loop (138 bubbles, of which 115 make contact). Its composition and distribution of contacting residues also differs. The Nb H2 contains fewer aromatic residues (Trp, Phe, Tyr) than the Ab H2- only 126 compared to 200—of which 67% make antigen contact compared to 81% in Abs. The most frequent antigen-contacting residues in Nb H2 loops are small residues—18% Ser and 15% Thr; while Ab H2 loop contacts are 15% Tyr, 13% Asn and 13% Ser.

The Nb H3 loop region contributes a total of 1353 antigen-contacting residues, 46% of all Nb:antigen contacts in the data set. This compares to only 865, or 38% of Ab:antigen contacts for the Ab H3 loop region. The Nb H3 uses a much greater diversity of residues (340 bubbles, of which 306 make antigen contact, compared to 252 in Abs, of which 195 contact the antigen). We note that the relatively conserved ‘YDY’ motif at the C-terminus of the Nb H3 loop is a particularly notable feature (labelled ‘Y2’) (Sircar *et al.*, 2011).

We previously showed that Nb loops are more structurally diverse than Ab loops, as measured by pairwise RMSD calculations (Mitchell and Colwell, 2018). In accordance with this observation, the PyIgClassify database (Adolf-Bryfogle *et al.*, 2015) finds that considerably fewer Nb structures have classifiable H1 and H2 loop conformations (77 H1, 111 H2), compared to the Ab data set (149 H1, 141 H2). Of the classifiable conformations, the most frequent classes in the Ab data set are H1-13-1 (83%), and H2-10-1 (50%). This represents far greater structural homogeneity than the Nb data set, where the most frequent classes are H1-13-1 (26%) and H2-10-2 (26%). Deviation from consensus loop conformations in Nbs is likely related to the variety of paratope residues sampled, and the greater diversity of structural segments used by Nb paratopes to contact the antigen.

### Residue interactions across the interface

**Fig. 6:**
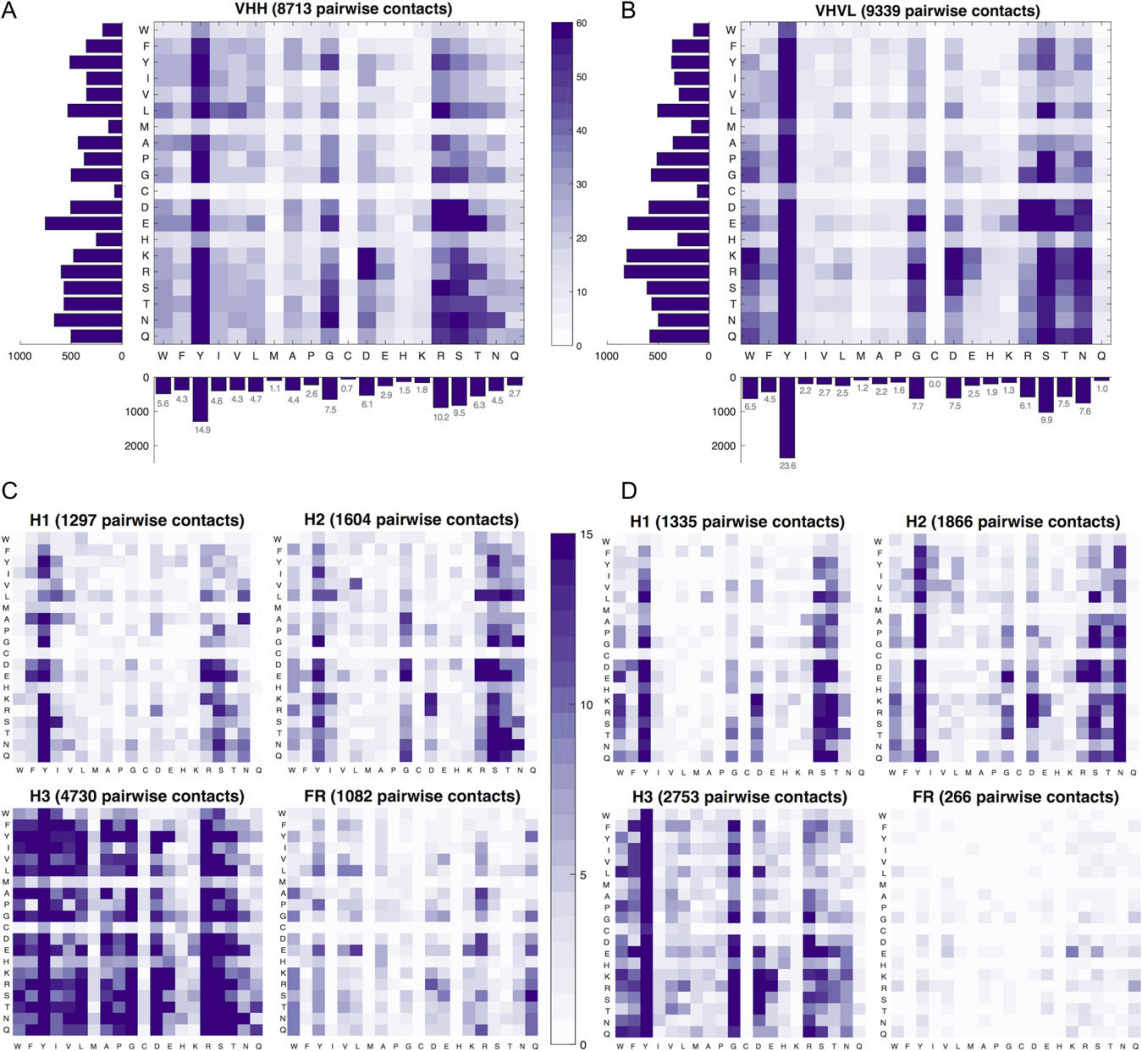
Heatmaps of the frequency of pairwise residue–residue contacts across Nb:antigen (left) and Ab:antigen (right) interfaces. Larger heatmaps (**A**) and (**B**) represent the frequency of each pairwise contact type across the entire full-length alignments, with high-frequency pairings coloured purple, and low-frequency pairings coloured white. Contacts contributed from the Nb or Ab are plotted along *x*-axis and contacts contributed from antigen are plotted along the *y*-axis. Histograms show the distribution of contacting residue type contributed by the two halves of the interface, with percentages annotated for the Nb/Ab side of the interface. Smaller heatmaps (**C**) and (**D**) represent a breakdown of pairwise residue–residue contact frequencies contributed by structural segments H1, H2, H3 and all four FRs combined. Total number of pairwise contacts represented by each plot is given in the titles. Equivalent plots for VH and VL domains are in Supplementary Fig. S5, data available at *PEDS* online.

We next examine pairwise residue–residue interactions across Nb:antigen interfaces. In Fig. 6, we plot the frequencies of all 200 possible pairwise residue–residue interactions, summed across each set of 156 structures. Note that single residues may be counted more than once if they are involved in multiple interchain pairwise interactions in a single co-crystal structure. Equivalent heatmaps for individual VH and VL alignments can be found in Supplementary Fig. S5, data available at *PEDS* online. The heatmaps reveal interesting differences between Ab:antigen and Nb:antigen interfaces. Overall, we find that Nb VHH domains make more pairwise contacts (8713) than VH domains (6220), but fewer than VHVL units (9339). Key differences include a greater contribution from Arg residues (10.2%) in Nbs, and more contacts involving the hydrophobic residues Ile, Val and Leu. Previous analyses of Ab:antigen interactions report an asymmetry in the types of residues and interactions used across the interface (Ramaraj *et al.*, 2012; Kunik *et al.*, 2012a; Sela-Culang *et al.*, 2013; Peng *et al.*, 2014; Robin *et al.*, 2014), and our data set shows that this trend extends to Nb:antigen interfaces. Nb paratopes are somewhat less dominated by antigen-contacting Tyr residues than Ab paratopes (23.6% of pairwise contacts in Abs, 14.9% in Nbs); however, in common with Ab paratopes small hydrophilic residues Gly and Ser play an important role. For both classes, the epitopes are characterized by a much more uniform distribution of residue types. Supplementary Fig. S6, data available at *PEDS* online, groups contacts into seven classes based on their paratope residue, revealing that Nbs use half the number of aromatic to positively charged interactions (often indicative of pi–cation interactions) that Abs do, while making greater use of hydrophobic and small residues. This observation could suggest an increased exploitation of entropic forces by Nb:antigen interfaces. We find that small residues frequently make contact with Glu, Asn, Asp, Ser, Thr and Tyr on the antigen surface.

We next split the contact frequencies according to the structural segment from which they originate. We find that 54.3% of Nb pairwise contacts involve the H3 loop, compared to 44.3% in the Ab VH set. Fewer contacts in total are contributed by Nb H2 loops (1604) than Ab H2 loops (1866). The Nb H2 loop contacts include many polar–polar interactions between Ser and Thr, particularly with negatively charged antigen surface residues Asp and Glu. The additional contacts mediated by Arg and hydrophobic residues in the Nb set are contributed predominantly from the H3 loop region, which has a vastly more varied distribution of contacts than the equivalent plot generated from the Ab pairwise contacts. Interactions that stand out as particularly frequent in Nbs include R-E, Y-E, Y-R, Y-N, D-R, G-N and G-Y. This suggests that charged interactions and aromatic interactions play a significant role in Nb:antigen interactions.

### Global interface properties

A striking finding is that Nbs make nearly as many pairwise contacts as the VHVL unit, despite being half the size. To investigate further, we normalize the total number of contacts by the total number of sequence positions (Table 1). Overall, Nb residues have an average contact density of 0.454 contacts per residue. This is 1.7 times greater than that of VHVL residues (0.262), and 1.4 times greater than that of VH residues (0.332). Notably, the contact density across Nb H3 loops is 2.009—considerably greater than the equivalent contact density for VH H3 loops (1.503), especially given that this calculation accounts for the effect of longer H3 loops in Nbs.

**Table 1.** Calculated interface properties for VHH, VHVL, VH and VL data sets

	VHH	VHVL	VH	VL
Contact density, all residues	0.454	0.262	0.332	0.184
Contact density, H1	0.825	–	0.843	0.450 (L1)
Contact density, H2	1.057	–	1.212	0.290 (L2)
Contact density, H3	2.009	–	1.503	0.555 (L3)
Mean paratope surface area (∆ASA), Å^2^	768.5 ± 201.0	833.8 ± 259.6	547.8 ± 199.6	286.0 ± 149.2
Mean shape complementarity statistic, S_C_	0.72 ± 0.07	0.70 ± 0.08	0.70 ± 0.08	–

Contact densities may be interpreted as the average ‘number of pairwise contacts made per residue’ and are calculated as described in the text.

We report that the Nb paratopes in this data set have a mean surface area of 768.50 ± 201.0 Å^2^; larger than the Ab VH paratopes (547.8 ± 199.6 Å^2^) and not a great deal smaller than VHVL paratopes (833.8 ± 259.6 Å^2^). A two-sample *t*-test reveals that the difference in mean paratope surface area between Nb and Ab VHVL paratopes is significant at the 5% significance level (*P* = 0.0135). Given that Nbs have smaller paratope surface areas than Abs, and higher contact densities, we might expect tighter shape complementarity to antigens. We find that Nbs in this data set have an average shape complementarity statistic (S_C_) of 0.72 ± 0.07. This is slightly, but significantly different to that of Ab VH domains (0.70 ± 0.08) and Ab VHVL units (0.70 ± 0.08), according to a two-sample *t*-test at the 5% significance level (*P* = 0.0031 or 0.0043, respectively). Previous work has shown that the S_C_ statistic can be artificially reduced in low-resolution structures (Cohen *et al.*, 2005; Kuroda and Gray, 2016), and we report a correlation between S_C_ and crystal structure resolution in both data sets (Supplementary data available at *PEDS* online, Fig. S7). If we restrict calculation of mean S_C_ to high-resolution crystal structures (resolution ≤2.5 Å), we obtain slightly higher statistics of 0.75 ± 0.04 over 85 Nb:antigen structures, and 0.73 ± 0.06 over 66 VHVL:antigen or VH:antigen structures.

## Conclusion

Nbs are single-domain Abs that show considerable promise for applications ranging therapeutic, diagnostic and molecular research spaces. Despite clear homology to VH domains, subtle structural differences suggest that there may be differences in the mechanisms used to determine interaction specificity. In this paper, we analyse the antigen-contacting residues from two sets of Nb:antigen and Ab:antigen co-crystal structures, to probe how paratopes are constructed and how they differ between the two classes of Ab. Three key differences between Nb and Ab paratopes emerge, which help to explain how Nbs achieve antigen binding with a single domain. First, there is much less consensus in Nb paratopes than Ab paratopes, both in terms of the type of residue and residue positions used, and the type of contacts made across the interface. Second, while Abs typically bind the antigen using the three sequence-variable loops, individual Nb paratopes can be constructed from many different combinations of surface patches, meaning the functional distinction between FR and loop positions is far less clear in Nbs. Finally, the H3 loop is more dominant in mediating antigen interactions than the other two loops, with almost one-third of Nbs not contacting the antigen via the H1 or H2 loops.

The finding that Nb paratopes have less consensus in their paratopes is most clearly shown by Fig. 3. The contact profile bar graphs show the majority of Abs bind the antigen via the three loops, without any FR contacts, in agreement with previous studies (Kunik *et al.*, 2012a,b). In contrast, Nbs sample a broader range of contact profiles. This finding, together with the contact propensity plot in Fig. 4 shows that Nb paratopes are drawn from a more diverse set of structural segments and aligned positions than Ab paratopes. These findings likely reflect the greater solvent-exposed surface area in Nbs, which do not need to maintain an interface with a cognate VL domain.

The lack of consensus in Nb paratopes translates to a blurring of the functional distinction between loop and FR positions found in Abs. The scatter plots in Fig. 5 show a clear distinction between specificity-determining and scaffold positions for Abs; in Nbs, alignment positions are more evenly distributed along the gradient between ‘scaffold’ and ‘specificity-determining’ clusters. This finding challenges the binary classification of residues used by classical Ab sequence-numbering schemes into loops and FR, considered analogous to ‘paratope’ and ‘non-paratope’. Many Nb H1 and H2 loop positions have intermediate levels of contact propensity and sequence variability, suggesting that they are not always involved in determining specificity. A considerable number of aligned positions are contained within this cluster and together represent a large amount of the equivalent surface patch typically used by Ab paratopes. This region of ambiguity has implications for computational docking of Nbs to antigens, while Ab docking is typically restrained by the selection of the expected paratope residues, which are equivalent to the three loop regions; in Nbs, a wider range of binding orientations should be considered.

Finally, our analysis shows there is substantial difference in the relative contribution to antigen binding from the three loops in Abs and Nbs. This finding is related to the reduction in paratope consensus, and the blurred distinction of specificity-determining and non-specificity-determining positions. In Abs, positions corresponding to the three variable loops have high contact propensity and high sequence variability, which suggests that these loops make similar contributions to determining specificity. In Nbs however, positions with high contact propensity and sequence variability are concentrated in the H3 loop region of the alignment. It has been previously established that Nb H3 loops are longer and more variable than their Ab counterparts (Muyldermans, 2013; Mitchell and Colwell, 2018), but for the first time we demonstrate here that they are also more frequently in contact with the antigen than the H1 or H2 loops; in terms of the number of H3 residues that make antigen contacts and also in terms of the number and diversity of pairwise residue–residue contacts. Each Nb H3 residue makes an average of 0.5 more pairwise contacts compared to those in Ab H3 loops. Furthermore, we note that H3 is the only loop involved in the paratope in all Nb structures. Taken together, these observations suggest that the H3 loop dominates Nb interaction specificity.

The lack of consensus among Nb paratopes—in terms of both combinations of structural segment used to contact the antigen, position/residue usage and contacts made—is a key distinguishing feature of Nbs compared to Abs. This suggests that the development of sequence-based paratope prediction methods such as ProABC (Olimpieri *et al.*, 2013) and Paratome (Kunik *et al.*, 2012a) will be more challenging for Nbs, since these methods make predictions based on Ab-specific paratope features, such as the three-loop paratope consensus. The finding that individual Nb paratopes draw from different combinations of structural segments is novel and is further reflected in the finding that the distinction between paratope and non-paratope sequence positions in general is less clear than in Ab VH domains. These observations, combined with our finding that the Nb H3 domain is the only structural segment that is always contained in the paratope, have important implications for Nb engineering and design, suggesting that attention should be concentrated on the Nb H3 loop and the interactions that it participates in. In summary, we present here the first comprehensive analysis of Nb:antigen interactions, revealing key differences in the way Nb and Ab paratopes are constructed, which has implications for the way we understand, model and engineer these single-domain Abs.

## Supplementary Material

Supplementary Data
