# Supplementary material for: Analysis of nanobody paratopes reveals greater diversity than classical antibodies
Source: Protein Eng Des Sel. 2018 Jul 25;31(7-8):267–75. doi: 10.1093/protein/gzy017 (PMC6277174; doi:10.1093/protein/gzy017)
Supplement: Supplementary Data [file gzy017_supplementary_information.pdf]

Supplementary Information:

Analysis of nanobody paratopes reveals greater diversity than classical antibodies

Laura S. Mitchell[1], Lucy J. Colwell[1]

[1]Department of Chemistry, University of Cambridge, Lensfield Road, Cambridge, CB2 1EW, UK

This work was supported by the Biotechnology and Biological Sciences Research Council [Award 1501548 to L.S.M.] and the Research Executive Agency [Grant Number: 631609 to L.J.C]

FIGURE S1 Full 161-position sequence alignment of 156 Nbs, followed by reduced 126-position alignment. Framework regions shown in grey, H1-3 in blue, green and red. Nb alignment positions with greater than 85% gaps are shown in yellow. These positions are excluded in the reduced 126-position Nb alignment, which is used for Figs. 4, 5, S3 and S5.

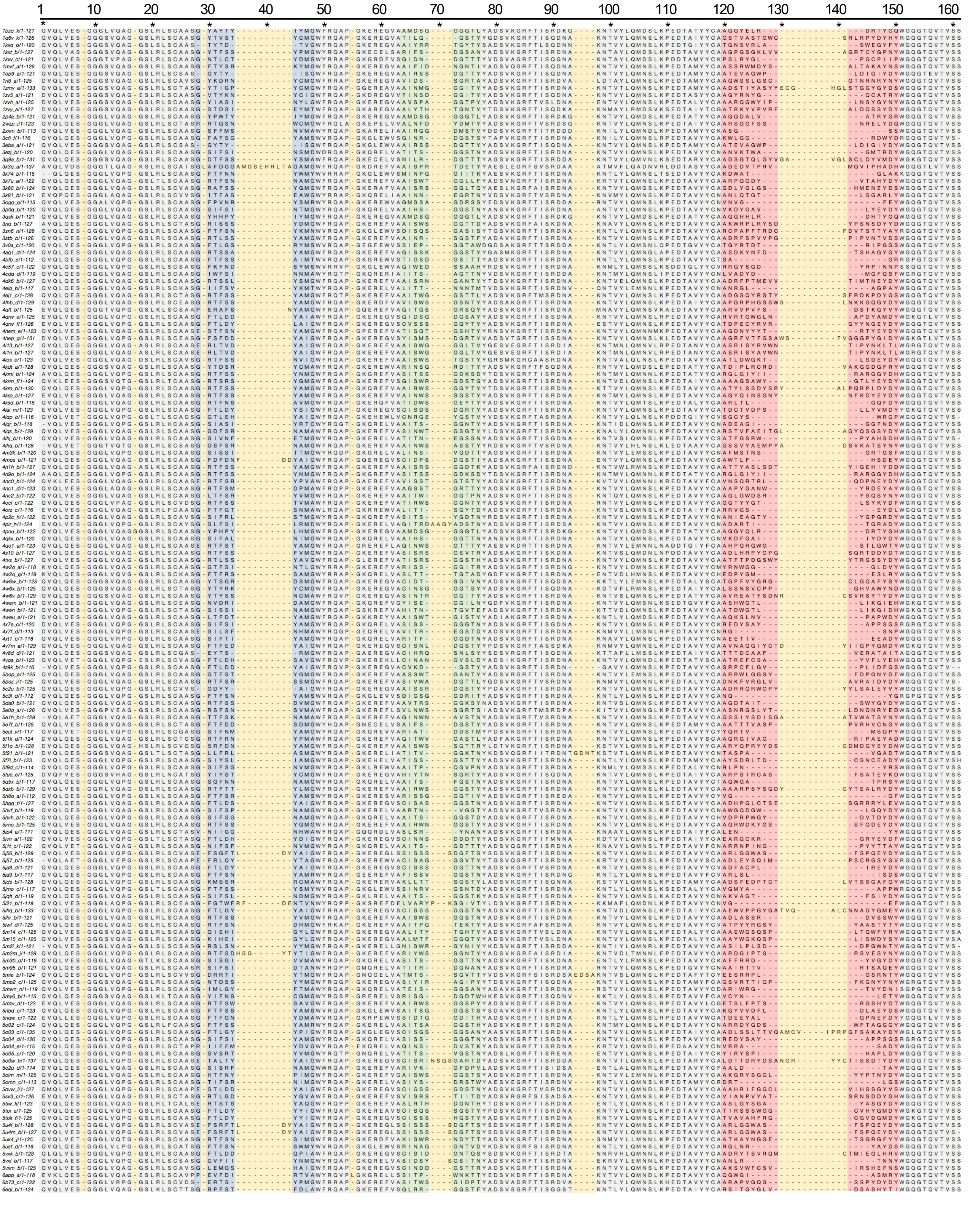

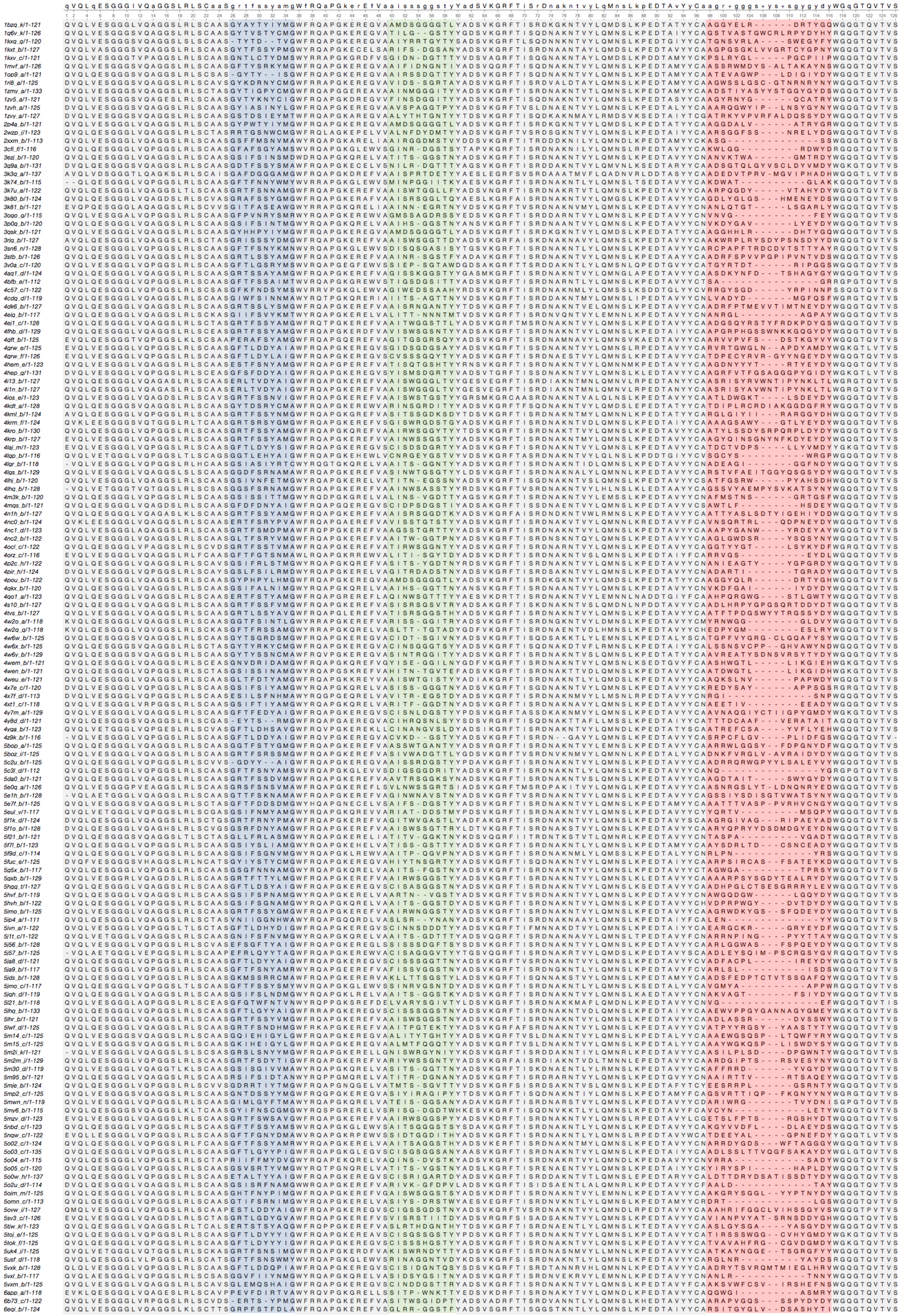

FIGURE S2 Full 161-position sequence alignment of 156 Ab VH sequences, followed by reduced 126-position alignment. Framework regions shown in grey, H1-3 in blue, green and red. Positions in yellow are equivalent to those in the Nb alignment with greater than 85% gaps. These positions are excluded in the reduced 126-position Ab alignment, which is used for Figs. 4, 5, S3 and S5.

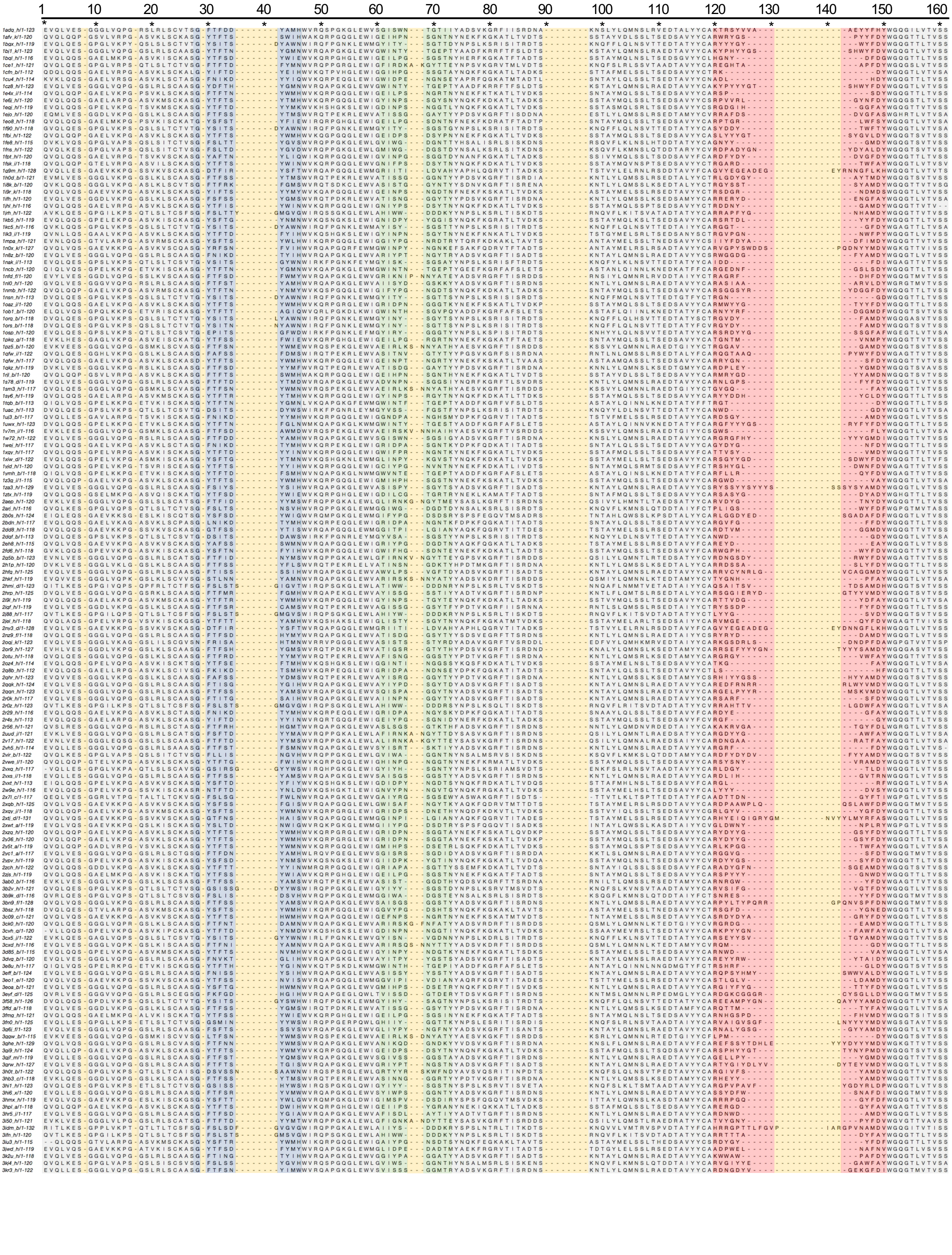

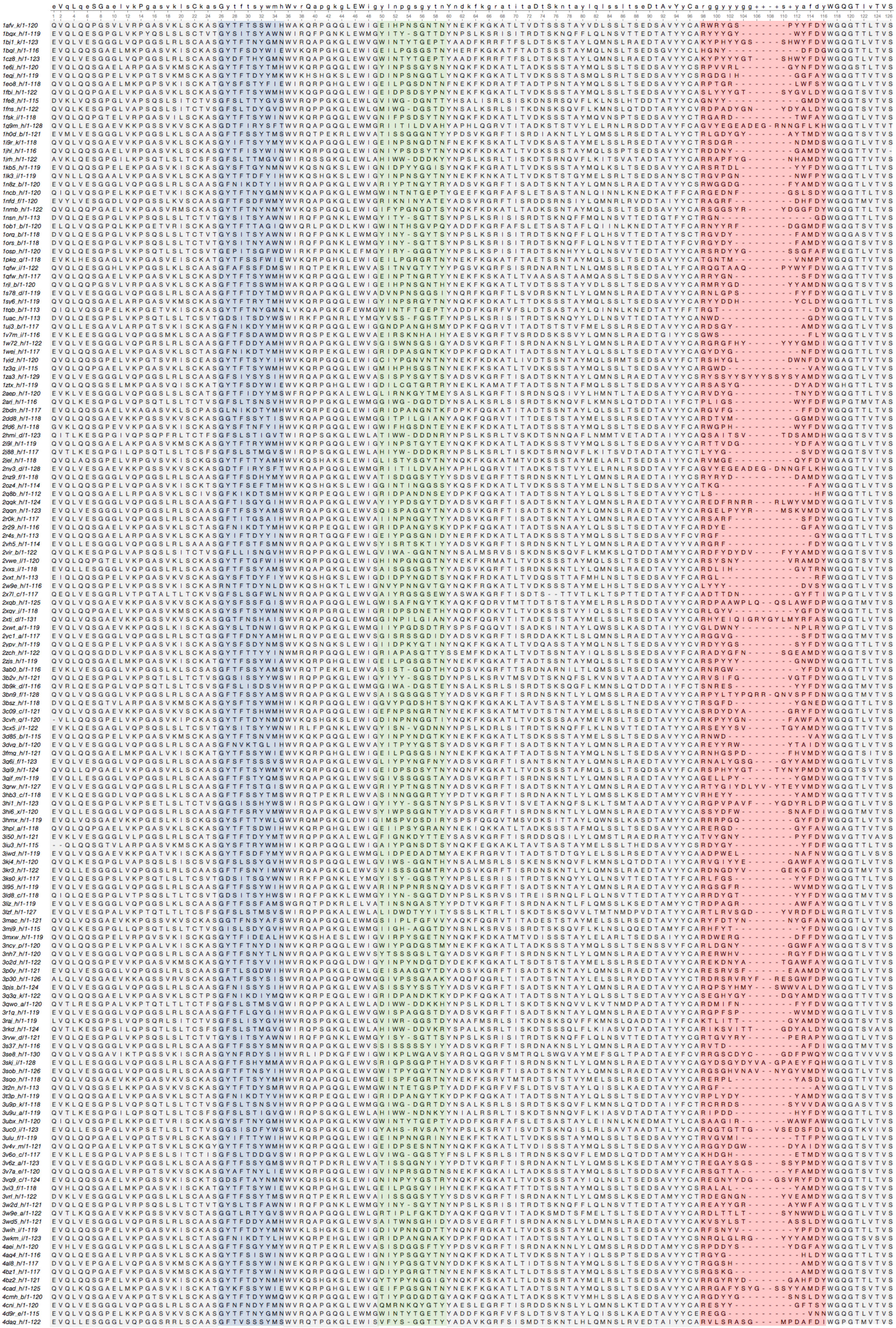

FIGURE S3 Distribution of solvent accessible surface areas observed for each aligned position in the VHH (A) and VH (B) datasets plotted as boxplots. The difference in mean SASA per position is plotted in Fig. 4C in the main text. Note that variation in SASA across the Ab FRs is greater than in Nb FRs as a result of greater sequence variation.

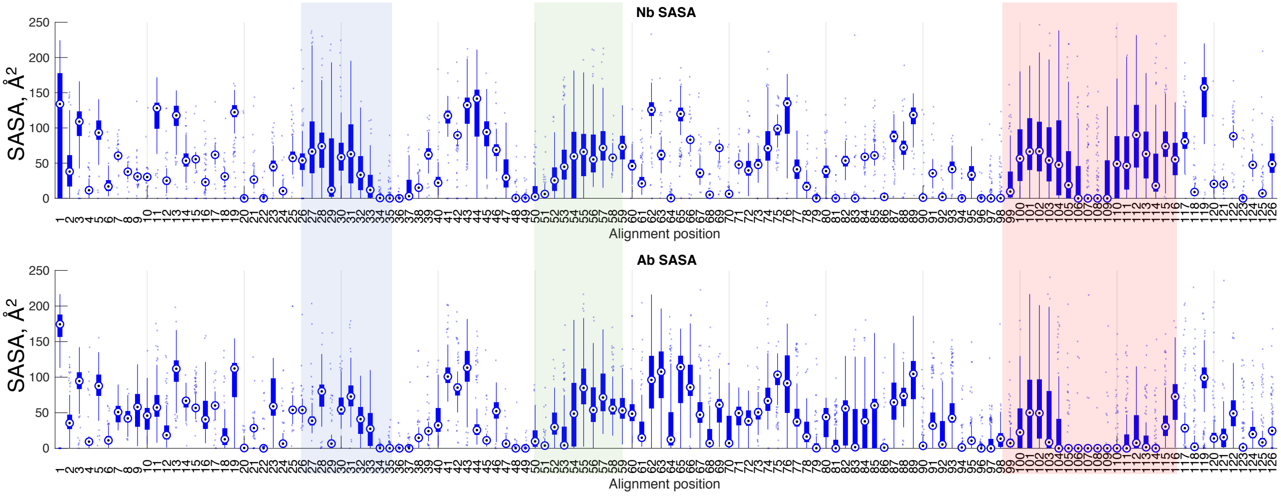

FIGURE S4 (A) Nb and (B) Ab sequence alignments and contact frequencies represented as bubble plots. The size of the grey points represents the frequency of the residue type at each alignment position, and the size of the red points represents the proportion of those residues which are within 5 Å of the antigen. Regions of interest are highlighted in yellow or purple, and referred to in the text by the annotations. The consensus sequence for each alignment is along the right hand side of the plot, with a capitalisation threshold at 90%, and '+' indicates positions with multiple modal residues.

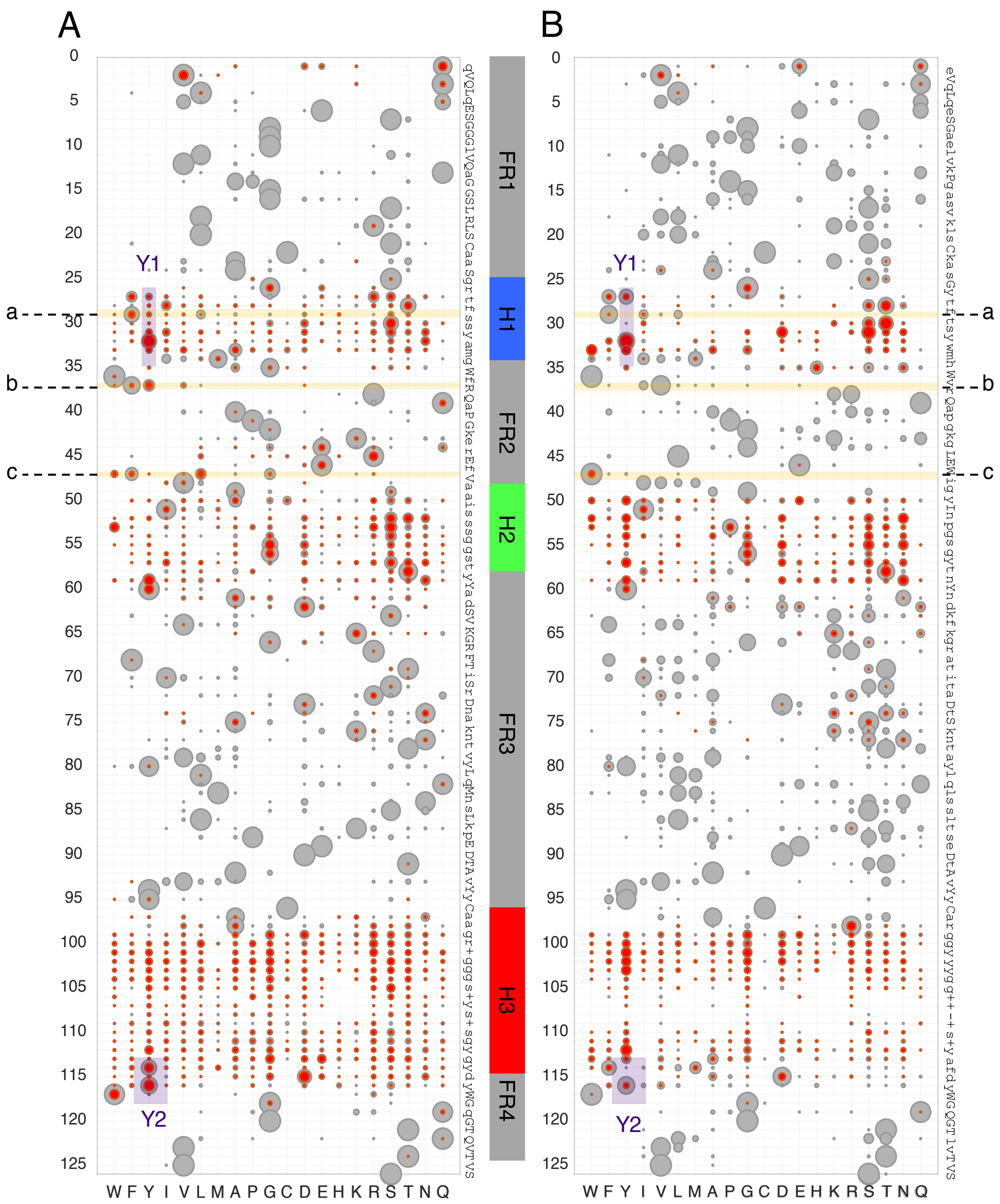

A
B
FR1
H1
FR2
H2
FR3
H3
FR4
Y1
Y2
a
b
c
W F Y I V L M A P G C D E H K R S T N Q

FIGURE S5 Heatmaps representing the frequency of pairwise residue-residue contacts across 156 Ab VH:antigen (A) and VL:antigen interfaces (B &C). Larger heatmaps (A &B) represent the frequency of each pairwise contact type across the entire full-length alignments, with high frequency pairings coloured purple, and low frequency pairings coloured white. Contacts contributed from Ab VH and VL domains are plotted along x-axis and contacts contributed from antigen are plotted along the y-axis. Histograms show the distribution of contacting residue type contributed by the two halves of the interface. Smaller heatmaps (C) represent a breakdown of pairwise residue-residue contact frequencies contributed by structural segments L1, L2, L3 and all four FRs combined. Total number of pairwise contacts represented by each plot is given in the titles.

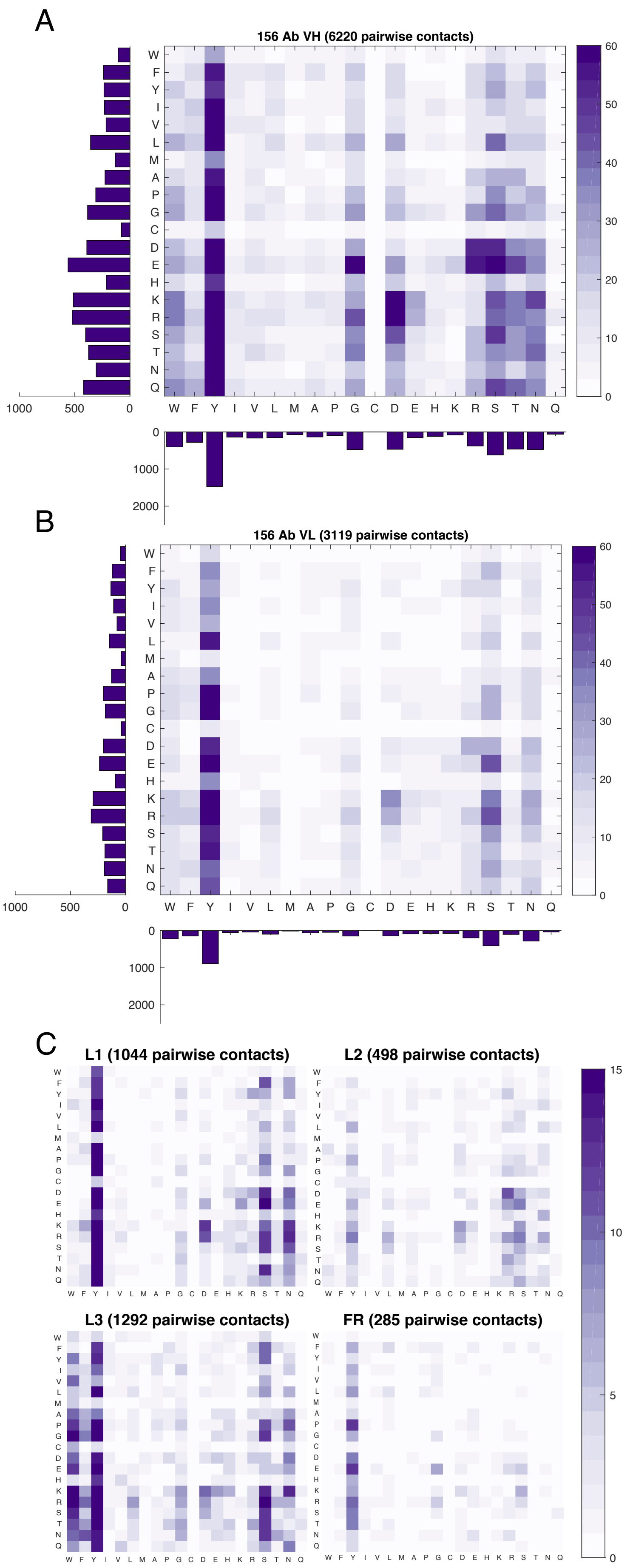
A
156 Ab VH (6220 pairwise contacts)
W F Y I V L M A P G C D E H K R S T N Q
1000 500 0
0 1000 2000
0 10 20 30 40 50 60
B
156 Ab VL (3119 pairwise contacts)
W F Y I V L M A P G C D E H K R S T N Q
1000 500 0
0 1000 2000
0 10 20 30 40 50 60
C
L1 (1044 pairwise contacts)
L2 (498 pairwise contacts)
L3 (1292 pairwise contacts)
FR (285 pairwise contacts)
W F Y I V L M A P G C D E H K R S T N Q
0 5 10 15

FIGURE S6 Pairwise contact heatmaps where contacts contributed by Nb VHH (A), Ab VHVL (B), Ab VH (C) and Ab VL paratopes (D) are grouped into seven classes.

Paratope residues are clustered into the following groups: Aromatic (FYW), Hydrophobic (VMILC), Small (SGAP), Negative (DE), Polar (QTN), Favoured positive (RH) and Disfavoured positive (K). Frequency of pairwise residue-residue contact types are coloured from white (infrequent) to purple (frequent), and the percentage each group of residues contributes to the total number of contacts is annotated.

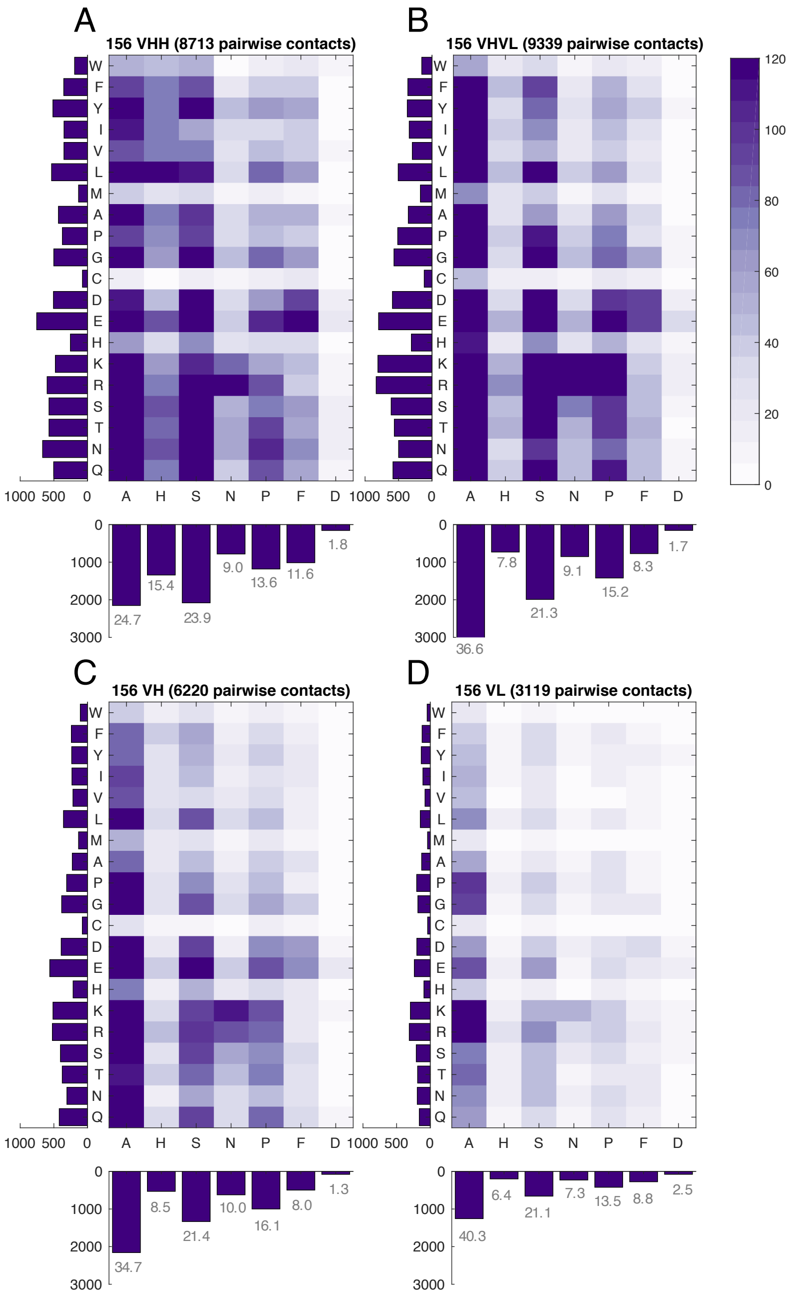

FIGURE S7 Scatterplots showing the correlation between shape complementarity statistic S_C and crystal structure resolution.

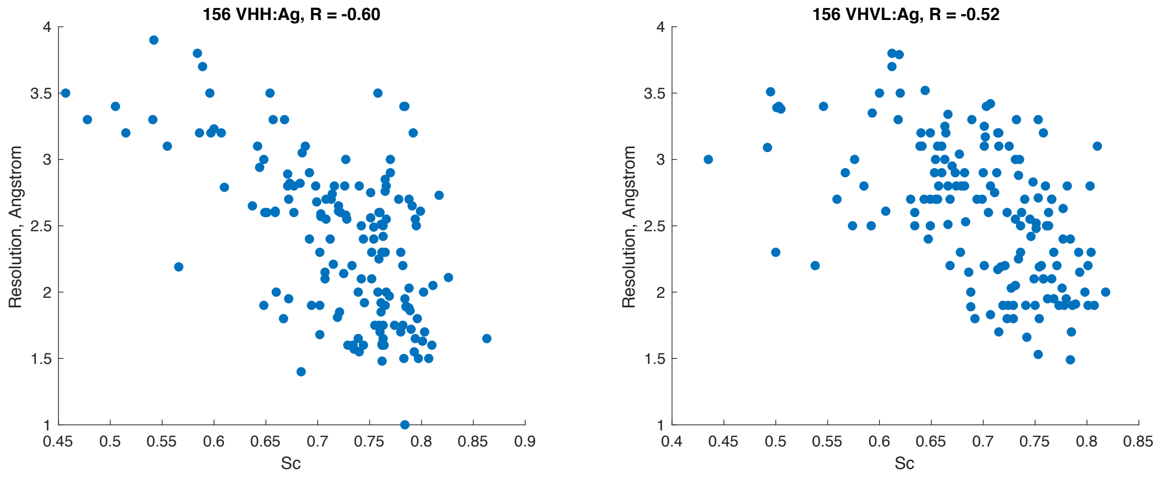

TABLE S1 Nanobody PDB files and Chain IDs. All structures were used as deposited in the PDB with exception to 4AQ1 and 4W6W, for which symmetry operations x,y-1,z and -y,x-y-1,z-1/3 were carried out on the respective antigen chains to obtain biologically relevant interfaces. These transformations were selected since they have the highest Complexation Significance Scores (CSS) of all crystal interfaces listed for those pdb files in the PDBePISA (Proteins, Interfaces, Structures and Assemblies) database.

Nanobodies

PDB	VHH	Ag	PDB	VHH	Ag	PDB	VHH	Ag
1BZQ	K	D	4LGP	B	A	5HGG	T	A
1G6V	K	A	4LGR	B	A	5HVF	B	A
1KXQ	G	B	4LGS	B	A	5HVH	B	A
1KXT	B	A	4LHJ	B	A	5IMO	B	A
1KXV	D	B	4LHQ	B	A	5IP4	B	D
1MVF	A	ED	4M3K	B	A	5IVN	A	B
1OP9	A	B	4MQS	B	A	5J1T	C	AB
1RI8	A	B	4N1H	B	A	5J56	B	A
1ZMY	A	L	4NC0	B	A	5J57	B	A
1ZV5	A	L	4NC1	C	A	5JA8	D	C
1ZVH	A	L	4NC2	B	A	5JA9	B	D
1ZVY	A	B	4OCL	C	B	5JDS	B	A
2P4A	B	A	4ORZ	C	B	5JMO	D	B
2WZP	D	B	4P2C	G	BA	5JQH	C	A
2XXM	B	A	4PIR	H	BC	5L21	B	A
3CFI	I	H	4POU	B	A	5LHQ	B	A

3EZJ	B	A
3G9A	B	A
3K3Q	A	B
3K74	B	A
3K7U	A	C
3K80	A	C
3K81	B	C
3OGO	G	B
3P0G	B	A
3QSK	B	A
3RJQ	B	A
3SN6	N	AB
3STB	B	C
3V0A	C	A
4AQ1	B	A
4FHB	D	A
4C57	C	A
4CDG	D	B
4DK6	B	C
4EIG	B	A
4EJ1	C	B
4BFB	E	B
4GFT	B	A
4GRW	E	CD
4GRW	F	CD
4HEM	F	CA
4HEP	G	A
4I13	B	A
4I1N	B	A
4IOS	E	AB
4KDT	A	D
4KML	B	A
4KRM	H	G
4KRO	B	A
4KRP	B	A
4LAJ	I	BC
4QKX	B	A
4QO1	A	B
4S10	A	C
4TVS	b	B
4W2O	A	B
4W2Q	G	H
4W6W	B	A
4W6Y	B	A
4W6X	B	A
4WEM	B	A
4WEN	B	A
4WEU	E	A
4X7E	C	A
4X7F	C	A
4XT1	C	A
4Y7M	A	C
4Y8D	D	B
4YGA	B	A
4Z9K	B	A
5BOP	A	B
5BOZ	H	B
5C2U	B	A
5C3L	D	CAB
5DA0	B	A
5E0Q	A	B
5E1H	B	A
5E7F	C	I
5EUL	V	YE
5F1K	D	B
5F1O	B	A
5F21	B	A
5F7L	D	C
5F9D	C	A
5FUC	V	AD
5G5X	B	A
5GXB	B	A
5LHR	B	A
5LWF	C	A
5M14	C	A
5M15	C	A
5M2I	K	D
5MP2	D	B
5M30	E	A
5M95	B	A
5MJE	B	A
5M2M	J	I
5MWN	N	A
5MY6	B	A
5MZV	D	A
5NBD	C	BA
5NQW	C	B
5O02	C	A
5O03	D	B
5O04	E	B
5O05	C	B
5O0W	H	D
5O2U	D	C
5OJM	M	C
5OMN	C	B
5OVW	I	B
5SV3	A	B
5TJW	K	A
5TOJ	D	BA
5TOK	E	BA
5UK4	j	J
5USF	D	A
5VXK	B	A
5VXL	B	A
5VXM	B	A
6APP	A	B
6B73	C	B
6EQI	B	CA

TABLE S2 Antibody PDB files and Chain IDs.

Antibodies

PDB	VH VL	Ag
1AFV	KM	B
1BGX	HL	T
1BJ1	KJ	V
1BQL	HL	Y
1CZ8	HL	W
1E6J	HL	P
1EGJ	HL	A
1EO8	HL	A
2JEL	HL	P
2NY3	DC	A
2NZ9	FE	B
2OZ4	HL	A
2Q8B	HL	A
2QQK	HL	A
2QQN	HL	A
2R0K	HL	A
3LD8	CB	A
3LIZ	HL	A
3LZF	HL	A
3MAC	HL	A
3MJ9	HL	A
3MXW	HL	A
3NCY	PS	BA
3NH7	HL	A

1FBI	HL	X	2R29	HL	A	3O2D	HL	A
1FE8	HL	A	2R4S	HL	A	3P0Y	HL	A
1FNS	HL	A	2VH5	HL	R	3P30	HL	A
1FSK	IH	G	2VIR	BA	C	3PJS	BA	MN
1G9M	HL	G	2VWE	LJ	B	3Q3G	KJ	L
1H0D	BA	C	2VXS	IM	AB	3QWO	AB	C
1I9R	KM	B	2VXT	HL	I	3R1G	HL	B
1JHL	HL	A	2W9E	HL	A	3RAJ	HL	A
1JRH	HL	I	2X7L	CD	P	3RKD	HL	A
1KB5	HL	AB	2XQB	HL	A	3RVW	DC	A
1LK3	IM	B	2XQY	JK	E	3S37	HL	X
1N8Z	BA	C	2XTJ	DB	A	3SE8	HL	G
1NCB	HL	N	2XWT	AB	C	3SKJ	IM	F
1NFD	FE	B	2YC1	AB	C	3SOB	HL	B
1NMB	HL	N	2YPV	HL	A	3SQO	HL	A
1NSN	HL	S	2ZCH	HL	P	3T2N	HL	A
1OB1	BA	C	2ZJS	HL	Y	3T3P	HL	AD
1ORQ	BA	C	3AB0	BC	A	3U9P	KM	C
1ORS	BA	C	3B2V	HL	A	3U9U	AB	F
1OSP	HL	O	3B9K	DC	F	3UBX	HL	A
1PKQ	GF	J	3BN9	FE	A	3UC0	IM	B
1QFW	IM	B	3BSZ	HL	F	3ULU	FE	A
1QFW	HL	A	3C09	CB	A	3V4V	MN	D
1RJL	BA	C	3CVH	QR	MO	3V6O	CE	A
1S78	DC	A	3CX5	JK	E	3V6Z	AB	F
1SY6	HL	A	3D85	BA	C	3V7A	EH	A
1TQB	BC	A	3DVG	BA	YX	3VG9	CB	A
1UAC	HL	Y	3FMG	HL	A	3VI3	FE	B
1UJ3	BA	C	3G6J	FE	BA	3VRL	HL	C
1V7M	IM	X	3GI9	HL	C	3W2D	HL	A
1W72	HL	AC	3GJF	MK	DF	3W9E	AB	C
1WEJ	HL	F	3GRW	HL	A	3WD5	HL	A
1YJD	HL	C	3HB3	CD	B	3WIH	IM	B
1Z3G	IM	B	3HI1	HL	G	3WKM	IM	B
1ZA3	HL	R	3HI6	XY	B	4AEI	HL	A
1ZTX	HL	E	3HMX	HL	A	4AG4	HL	A
2AEP	HL	A	3HPL	AB	C	4AL8	HL	C
2ARJ	HL	Q	3I50	HL	E	4BZ1	HL	A
2BDN	HL	A	3IU3	HL	I	4BZ2	HL	A
2DD8	HL	S	3JWD	HL	A	4CAD	HG	I
2FD6	HL	U	3KJ4	HL	A	4CMH	BC	A
2HMI	DC	B	3KR3	HL	D	4CNI	HL	C
2I9L	HG	L	3KS0	KJ	A	4D9R	ED	B
2J88	HL	A	3L95	HL	Y	4DAG	HL	A

TABLE S3 Alignment position to AHo numbering index table. The nanobody consensus sequence is listed in the third column for reference – the capitalisation threshold is 90%, and '+' indicates positions with multiple modal residues. H1-3 loop positions used in this study are coloured blue, green and red.

Alignment numbering	AHo numbering	Nb Consensus
1	1	q
2	2	V
3	3	Q
4	4	L
5	5	q
6	6	E
7	7	S
8	9	G
9	10	G
10	11	G
11	12	l
12	13	V
13	14	Q
14	15	a
15	16	G
16	17	G
17	18	S
18	19	L
19	20	R
20	21	L
21	22	S
22	23	C
23	24	a
24	25	a
25	26	S
26	27	g
27	29	r
28	30	t
29	31	f
30	32	s
31	33	s
32	39	y
33	40	a
34	41	m
35	42	g
36	43	W
37	44	f
38	45	R
39	46	Q
40	47	a
41	48	P
42	49	G
43	50	k
44	51	e
45	52	r
46	53	E
47	54	f
48	55	V
49	56	a
50	57	a
51	58	i
52	59	s
53	60	s
54	61	s
55	65	g
56	66	g
57	67	s
58	68	t
59	69	y
60	70	Y
61	71	a
62	72	d
63	73	S

Aligment numbering	AHo numbering	Nb Consensus
64	74	V
65	75	K
66	76	G
67	77	R
68	78	F
69	79	T
70	80	i
71	81	S
72	82	r
73	83	D
74	84	n
75	85	a
76	86	k
77	87	n
78	88	t
79	89	v
80	90	y
81	91	L
82	92	q
83	93	M
84	94	n
85	95	s
86	96	L
87	97	k
88	98	p
89	99	E
90	100	D
91	101	T
92	102	A
93	103	v
94	104	Y
95	105	y
96	106	C
97	107	a
98	108	a
99	109	g
100	110	r
101	111	+
102	112	g
103	113	g
104	114	g
105	115	s
106	116	+
107	117	y
108	130	s
109	131	+
110	132	s
111	133	g
112	134	y
113	135	g
114	136	y
115	137	d
116	138	y
117	139	W
118	140	G
119	141	q
120	142	G
121	143	T
122	144	Q
123	145	V
124	146	T
125	147	V
126	148	S
